# t-Darpp overexpression in HER2-positive breast cancer confers a survival advantage in lapatinib

**DOI:** 10.18632/oncotarget.5311

**Published:** 2015-09-28

**Authors:** Jessica L. Christenson, Erin C. Denny, Susan E. Kane

**Affiliations:** ^1^ Department of Cancer Biology, Beckman Research Institute at City of Hope, Duarte, CA 91010, USA; ^2^ Current address: Department of Pathology, University of Colorado Denver, Aurora, CO 80045, USA; ^3^ Current address: Amgen, Thousand Oaks, CA 91320, USA

**Keywords:** t-Darpp, HER2, lapatinib, BIM, resistance

## Abstract

Drug resistance is a major barrier to successful cancer treatment. For patients with HER2-positive breast cancer who initially respond to therapy, the majority develop resistance within one year of treatment. Patient outcomes could improve significantly if we can find and exploit common mechanisms of acquired resistance to different targeted therapies. Overexpression of t-Darpp, a truncated form of the dual kinase/phosphatase inhibitor Darpp-32, has been linked to acquired resistance to trastuzumab, a front-line therapy for HER2-positive breast cancer. Darpp-32 reverses t-Darpp's effect on trastuzumab resistance. In this study, we examined whether t-Darpp could be involved in resistance to lapatinib, another HER2-targeted therapeutic. Lapatinib-resistant SKBR3 cells (SK/Lap^R^) showed a marked change in the Darpp-32:t-Darpp ratio toward a predominance of t-Darpp. Overexpression of t-Darpp alone was not sufficient to confer lapatinib resistance, but cells that overexpress t-Darpp partially mimicked the molecular resistance phenotype observed in SK/Lap^R^ cells exposed to lapatinib. SK/Lap^R^ cells failed to down-regulate Survivin and failed to induce BIM accumulation in response to lapatinib; cells overexpressing t-Darpp exhibited only the failed BIM accumulation. t-Darpp knock-down reversed this phenotype. Using a fluorescence-based co-culture system, we found that cells overexpressing t-Darpp formed colonies in lapatinib within 3–4 weeks, whereas parental cells in the same co-culture did not. Overall, t-Darpp appears to mediate a survival advantage in lapatinib, possibly linked to failed lapatinib-induced BIM accumulation. t-Darpp might also be relevant to acquired resistance to other cancer drugs that rely on BIM accumulation to induce apoptosis.

## INTRODUCTION

Trastuzumab, a humanized, monoclonal antibody targeted to HER2 (human epidermal growth factor receptor 2), is the principal treatment for patients with HER2-positive (HER2+) breast cancer. Although it can be effective initially, the majority of trastuzumab-responsive patients develop resistance within one year of treatment [1, 2]. Newer, second-line HER2-targeted therapies, such as the small molecule dual HER2/EGFR (epidermal growth factor receptor) inhibitor lapatinib, seem to be following a similar pattern of initial response followed by acquired resistance [3–5].

Resistance to HER2-targeted agents can develop through a variety of mechanisms. Resistant cells might shift their signaling to a compensatory receptor or signal transduction pathway or they might modulate genes involved in proliferation or survival [2, 6]. Because many different avenues are available, it is critical to understand how cells acquire resistance and evade therapeutic effects, with the goal of developing treatment strategies to target those pro-survival pathways. It would be particularly valuable to understand common mechanisms that might be responsible for resistance to multiple agents used against the same target.

t-Darpp is a protein that has been directly linked to acquired trastuzumab resistance in HER2+ breast cancer cells. Overexpression of endogenous t-Darpp has been reported in several independently-developed trastuzumab-resistant cell lines, and overexpression of exogenous t-Darpp is sufficient to confer resistance in otherwise trastuzumab-sensitive cells [7–10]. t-Darpp is a truncated variant of Darpp-32 (dopamine and cAMP-regulated phosphoprotein of 32 kDa), a protein that is well-characterized as a mediator of cell signaling in neuronal cells and which might function as a tumor suppressor and anti-metastatic protein in the context of cancer [8, 11–16]. t-Darpp was first discovered in gastric cancer patient samples and subsequently was found to be expressed in several types of adenocarcinoma including breast cancer [17, 18]. Its normal cellular function is not known, but it has been proposed as a putative oncogene, able to increase cellular growth and inhibit apoptosis in addition to conferring trastuzumab resistance [7–10, 17, 19–21]. Co-expression of exogenous Darpp-32 along with t-Darpp reverses the trastuzumab resistance phenotype mediated by t-Darpp [8]. This seems to suggest that Darpp-32 and t-Darpp have antagonistic roles in modulating the cellular response to trastuzumab, with t-Darpp acting as the pro-growth, pro-resistance form of the protein. The purpose of this study was to investigate whether t-Darpp could be involved in resistance to other HER2-targeted therapeutic agents such as the small-molecule inhibitor lapatinib.

## RESULTS

### Resistant breast cancer cell lines show a shift in the ratio of Darpp-32 to t-Darpp

**Figure 1:**
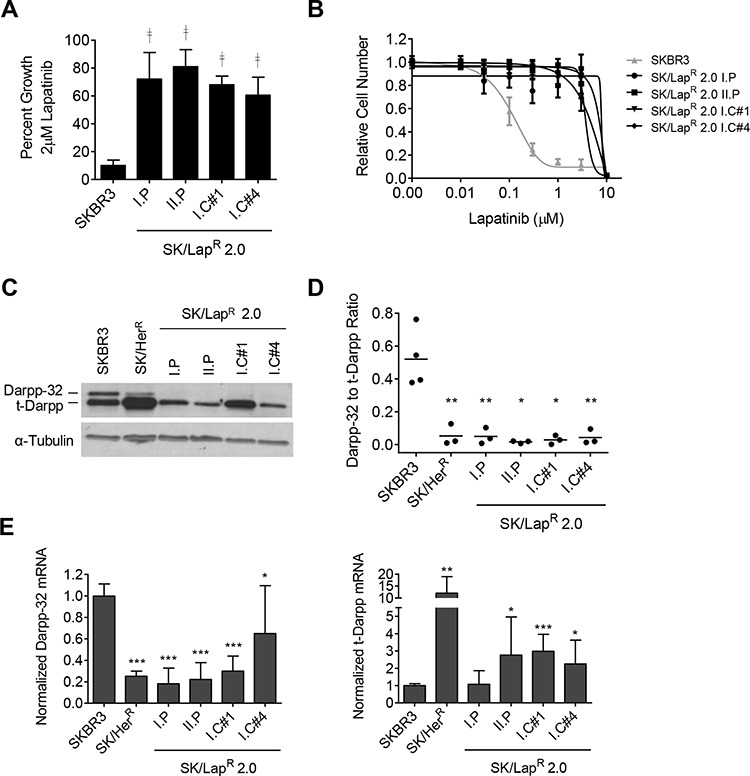
SK/LapR cells **A.** Cell growth in the indicated cell lines was measured by CellTiter-Glo luminescence assay after a 5-day exposure to 0.1% DMSO or 2 μM lapatinib. Data was normalized to the mean luminescence of DMSO-treated cells; mean ± standard deviation. **B.** Proliferation in lapatinib was quantified by SRB assay after 5-day exposure to either 0.1% DMSO or increasing concentrations of lapatinib. Data was normalized to the mean absorbance of DMSO-treated cells; mean ± standard deviation. **C.** Darpp-32 and t-Darpp protein levels were measured by Western analysis. α-Tubulin was used as a loading control. **D.** Western data was quantified using ImageJ software. Relative Darpp-32 and t-Darpp levels were calculated and each individual ratio from replicate experiments was plotted. The bar indicates the mean. **E.** Darpp-32 and t-Darpp mRNA levels were measured by SYBR Green quantitative RT-PCR. Data was normalized to GAPDH and SKBR3 expression levels; mean ± standard deviation. *p ≤ 0.05, **p ≤ 0.01, ***p ≤ 0.001, ^╪ ╪^*p* ≤ 0.0001 for each cell line compared to SKBR3 cells.

To determine if t-Darpp plays a role in lapatinib resistance, we first developed lapatinib-resistant cell lines. SKBR3 cells were treated continuously with increasing concentrations of lapatinib for 6–12 months until they were able to proliferate in 2 μM lapatinib (Fig. 1A). Multiple lapatinib-resistant cell lines (SK/Lap^R^) were derived, including two independently developed pooled populations (I.P and II.P) and two clonal lines originally isolated from the I.P selection before the colonies were pooled (I.C#1 and I.C#4). The lapatinib IC_50_s of the resistant cell lines were at least 25-fold higher, on average, than the IC_50_ for parental SKBR3 cells (Fig. 1B).

If t-Darpp is involved in lapatinib resistance, we would expect to see changes in its expression in SK/Lap^R^ cell lines, as we and others saw in cells selected for trastuzumab resistance [7–9, 19]. Because the relative levels of Darpp-32 and t-Darpp seem to be important in determining resistance, we examined both Darpp-32 and t-Darpp protein (Fig. 1C and 1D) and mRNA (Fig. 1E) in all resistant cells lines. Unlike trastuzumab-resistant SK/Her^R^ cells, which show a marked elevation of t-Darpp relative to SKBR3 cells, SK/Lap^R^ cells showed little to no change in t-Darpp protein levels, even though t-Darpp mRNA levels were moderately increased in several of the SK/Lap^R^ cell lines (Fig. 1C and 1E). Instead, all lapatinib-resistant cells exhibited a clear decrease in Darpp-32 protein and mRNA (Fig. 1C and 1E). In both SK/Her^R^ and SK/Lap^R^ cells, there was a significant decrease in the ratio of Darpp-32 to t-Darpp protein, relative to that seen in parental SKBR3 cells (Fig. 1D).

### t-Darpp overexpression does not confer lapatinib resistance

**Figure 2:**
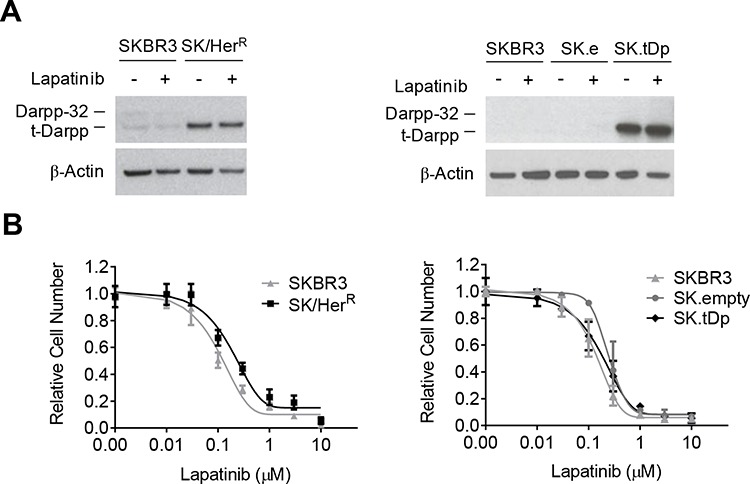
Lapatinib sensitivity in cell lines overexpressing t-Darpp SK/Her^R^ trastuzumab-resistant cells overexpress endogenous t-Darpp and SK.tDp cells overexpress stably transfected exogenous t-Darpp. SK.empty (SK.e) cells carry a stably transfected empty vector control. **A.** Darpp-32 and t-Darpp protein levels were measured by Western analysis in 0.1% DMSO (−) or 2 μM lapatinib (+) for 24 hours. β-Actin was used as a loading control. **B.** Proliferation in lapatinib was determined by SRB assay after 7-day exposure to either 0.1% DMSO or increasing concentrations of lapatinib. Data was normalized to the mean absorbance of DMSO-treated cells; mean ± standard deviation.

To determine if t-Darpp overexpression can confer lapatinib resistance, we examined lapatinib sensitivity in SK/Her^R^ cells that overexpress endogenous t-Darpp and in SK.tDp cells that stably overexpress exogenous t-Darpp introduced by cDNA transfection. Neither cell line showed a change in t-Darpp expression after 24-hour exposure to lapatinib (Fig. 2A), and the lapatinib IC_50_ was the same in SKBR3 and both of the cell lines that overexpress t-Darpp (Fig. 2B). These results are consistent with published reports suggesting no inherent cross-resistance between trastuzumab and lapatinib in both cell lines and patients [22, 23]. Furthermore, t-Darpp down-regulation in SK/Lap^R^ cells did not significantly alter sensitivity to lapatinib-mediated apoptosis (Supplementary Fig. 1), again suggesting that t-Darpp is not responsible for the lapatinib resistance phenotype in these cells.

### t-Darpp overexpression partially mimics the molecular resistance phenotype of SK/Lap^R^ cells

**Figure 3:**
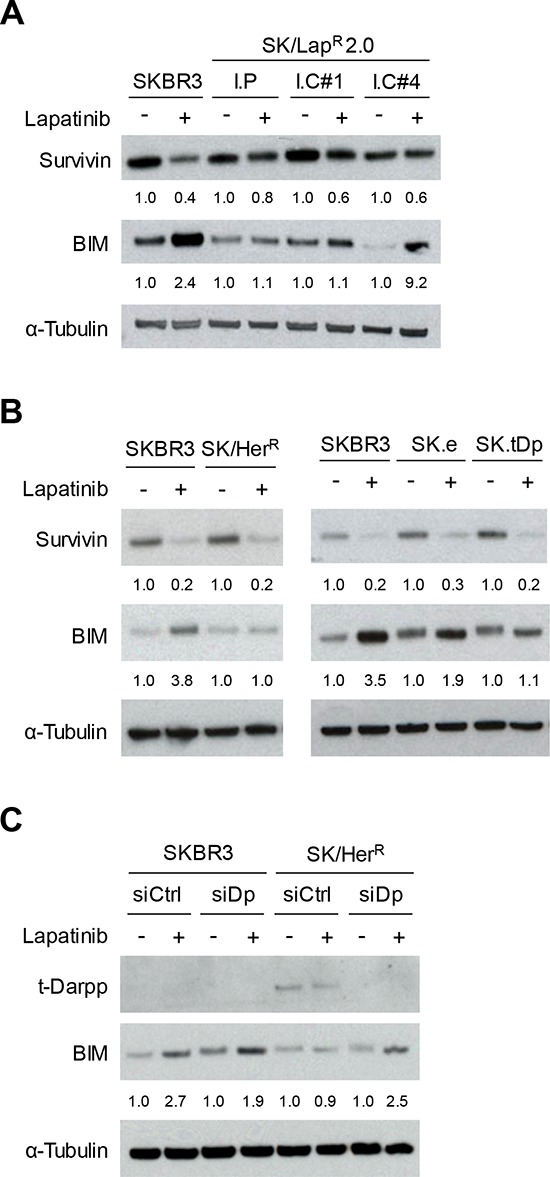
Survivin and BIM expression in response to lapatinib Western analysis of Survivin and BIM protein levels after 24 hour exposure to 0.1% DMSO (−) or 2 μM lapatinib (+) in lapatinib-resistant cells **(A),** cells overexpressing t-Darpp **(B),** or SK/Her^R^ cells transiently transfected with siRNA targeted to GFP (siCtrl) or Darpp-32/t-Darpp (siDp) for 72 hours **(C).** α-Tubulin was used as a loading control. Protein expression was quantified using ImageJ software. Data was normalized to α-Tubulin levels and expressed as the fold change in protein level after lapatinib treatment, relative to the DMSO control, for each cell line.

As a further analysis of SK/Lap^R^ cells, we looked at several molecular markers of signal transduction and cell survival in response to lapatinib (Supplementary Fig. 2A). We noted some key changes in the signaling status of SK/Lap^R^ relative to SKBR3 cells. SK/Lap^R^ cells appeared to have lower basal levels of total HER2, phosphorylated HER2 (pHER2) and phosphorylated EGFR (pEGFR) than SKBR3 cells, although both pHER2 and pEGFR were completely down-regulated by lapatinib in both SK/Lap^R^ and SKBR3 cells. In contrast, SK/Lap^R^ cells had fully or partially sustained levels of phosphorylated Akt (pAkt; protein kinase B) and phosphorylated ERK (pERK; extracellular signal-regulated kinase) after exposure to lapatinib, both of which were completely inhibited in SKBR3 cells (Supplementary Fig. 2A). Changes were also observed in the response of proteins essential for the induction of apoptosis by lapatinib. Lapatinib causes apoptosis by simultaneously down-regulating the anti-apoptotic protein Survivin and upregulating the pro-apoptotic protein BIM (Bcl2 homology domain 3(BH3)-only protein) [24]. We observed both of these responses in parental SKBR3 cells, but lapatinib no longer stimulated BIM nor down-regulated Survivin in SK/Lap^R^ cells (Fig. 3A). The SK/Lap^R^ 2.0 I.C#4 clone, unlike the other SK/Lap^R^ cells, did seem to induce BIM accumulation in response to lapatinib. However, this cell line expressed extremely low basal levels of BIM in the absence of lapatinib. Even after lapatinib exposure, BIM levels in clone 4 remained lower than those observed in SKBR3 cells exposed to lapatinib in parallel, consistent with the established theory that low BIM levels are associated with poor response to the drug [25].

To determine if cells overexpressing t-Darpp shared any of the aberrant molecular signaling observed in SK/Lap^R^ cells, we examined the same set of signaling and apoptotic proteins in SK/Her^R^ and SK.tDp cells. HER2 signal transduction in response to lapatinib was essentially the same in SK/Her^R^, SK.tDp and parental SKBR3 cells (Supplementary Fig. 2B and 2C). Likewise, lapatinib was still capable of suppressing Survivin in SK/Her^R^ and SK.tDp cells, as in SKBR3 cells (Fig. 3B). However, lapatinib failed to induce BIM accumulation in either SK/Her^R^ or SK.tDp cells, similar to what was observed in SK/Lap^R^ cells (Fig. 3B). Notably, t-Darpp down-regulation in SK/Lap^R^ cells did not affect lapatinib-mediated BIM induction, consistent with the earlier observation that t-Darpp down-regulation did not alter lapatinib-mediated apoptosis (Supplementary Fig. 1). We did observe a small but statistically significant effect of t-Darpp down-regulation on BIM and apoptosis levels in the absence of lapatinib (Supplemental Fig. 1), suggestive of some role for t-Darpp in regulating these effects without being responsible for conferring the full lapatinib resistance effect in SK/Lap^R^ cells.

The failure to induce BIM in cells that overexpress t-Darpp (SK.tDp and SK/Her^R^) suggests that t-Darpp is sufficient to mediate this effect in otherwise lapatinib-responsive SKBR3 cells. To determine if t-Darpp is necessary for the failed accumulation of BIM in SK/Her^R^ cells, we used siRNA to down-regulate t-Darpp in these cells (Fig. 3C). SK/Her^R^ cells transfected with control siRNA failed to induce BIM accumulation in response to lapatinib, as previously observed, whereas lapatinib-mediated BIM accumulation was rescued in cells transfected with Darpp-specific siRNA, to a degree comparable to that seen in parental SKBR3 cells (Fig. 3C). Since BIM upregulation is believed to be necessary for a full lapatinib-induced apoptotic response [24], these experiments suggest that t-Darpp overexpression is both sufficient to confer a partial lapatinib resistance phenotype (failed BIM accumulation) in SKBR3 cells and required for the failed BIM accumulation phenotype in SK/Her^R^ cells.

### t-Darpp overexpression promotes accelerated colony formation in lapatinib

Based on the previous observations that cells overexpressing t-Darpp failed to induce BIM accumulation, thus partially mimicking the molecular resistance phenotype of SK/Lap^R^ cells, we hypothesized that t-Darpp overexpression might prime cells to become fully resistant to lapatinib more quickly than cells with low t-Darpp levels. To investigate this possibility, we performed a preliminary experiment in which we examined changes in lapatinib sensitivity after one or two weeks of exposure to the drug. By two weeks, SK/Her^R^ and SK.tDp cells appeared to be less growth-inhibited by lapatinib than parental cells, although only the SK/Her^R^ cells reached statistical significance (*p* = 0.007; Supplementary Fig. 3).

To investigate more directly the role of t-Darpp in the development, or priming, of lapatinib resistance, we wanted to examine lapatinib sensitivity over a more prolonged exposure to the drug. To accomplish this, we developed a co-culture model in which we used fluorescently-tagged cell lines to track relative cell survival and colony formation over time. SKBR3 cells were stably transfected with a vector encoding EGFP (SKBR3.EGFP) while SK/Her^R^ and SK.tDp cells were stably transfected with a vector encoding mCherry (SK/Her^R^.mCherry and SK.tDp2A.mCherry, respectively). No changes in baseline Darpp-32 or t-Darpp expression (Supplementary Fig. 4A) or lapatinib sensitivity (Supplementary Fig. 4B) were observed in cells expressing EGFP or mCherry. Co-cultures of SKBR3.EGFP and SK/Her^R^.mCherry cells (1:1 ratio) were established and grown in the presence of either 0.6 μM or 1.0 μM lapatinib or DMSO control for five weeks. Cell survival and proliferation were tracked weekly via fluorescent cell imaging and flow cytometry. All data from lapatinib-treated cells was normalized to DMSO-treated cells to account for any inherent differences in growth between the co-cultured cell lines.

**Figure 4:**
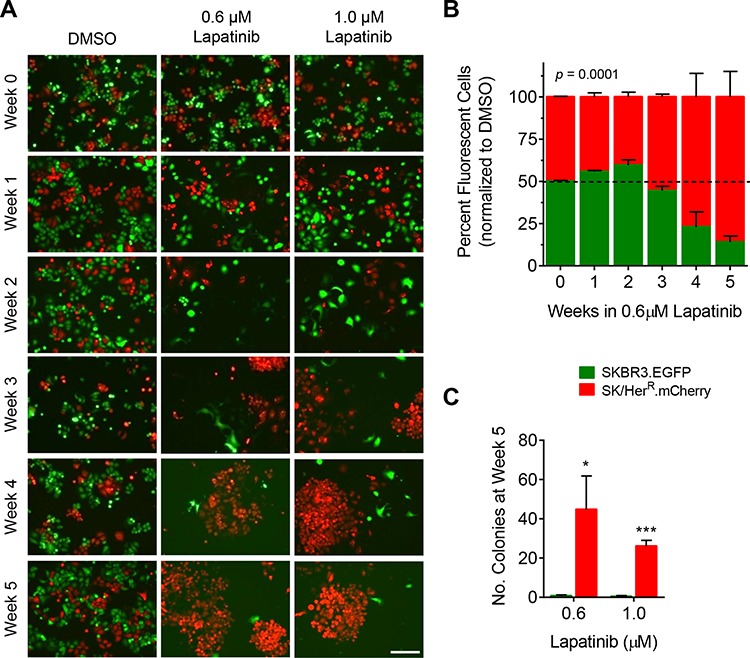
Colony formation by SK/Her^R^ cells exposed to lapatinib SKBR3 cells stably expressing EGFP (SKBR3.EGFP) and SK/Her^R^ cells stably expressing mCherry (SK/Her^R^.mCherry) were co-cultured at a ratio of 1:1 and continuously exposed to 0.1% DMSO, 0.6 μM lapatinib or 1.0 μM lapatinib for 5 weeks. DMSO-treated cells were split twice-weekly (1:4 dilution). Lapatinib-treated cells were grown without passaging. Each experiment was run in triplicate. **A.** Co-cultured cells were imaged weekly for fluorescence (10x magnification, scale bar = 200 μm). Shown are representative fields for each condition and time point. **B.** The percentage of EGFP-positive and mCherry-positive cells in each co-culture was quantitatively measured weekly via flow cytometry. Data was normalized to the mean percentage of EGFP-positive and mCherry-positive cells, respectively, in DMSO-treated co-cultures; mean ± standard deviation. **C.** For each co-culture the number of fluorescent colonies was counted after 5 weeks in lapatinib; mean ± standard deviation, *p ≤ 0.05, ***p ≤ 0.0001.

As expected, over the first two weeks of culturing, SKBR3 and SK/Her^R^ cells both underwent cell death in response to lapatinib (Fig. 4A). By weeks 3 and 4, however, a clear difference began to emerge, with SK/Her^R^ cells starting to form colonies but no apparent colony formation by SKBR3 cells (Fig. 4A). This was reflected in the flow cytometry measurements as a shift in the population towards the mCherry-positive cells (Fig. 4B, week 4). By week 5, the cultures were comprised mostly of mCherry-positive cells by flow cytometry (*p* = 0.0001; Fig. 4B) and there were significantly more mCherry-positive colonies present than EGFP-positive colonies (*p* = 0.011 for 0.6 μM lapatinib, *p* = 0.0001 for 1.0 μM; Fig. 4C). This suggests a clear survival advantage for mCherry-positive SK/Her^R^ cells, relative to SKBR3 cells, in lapatinib.

**Figure 5:**
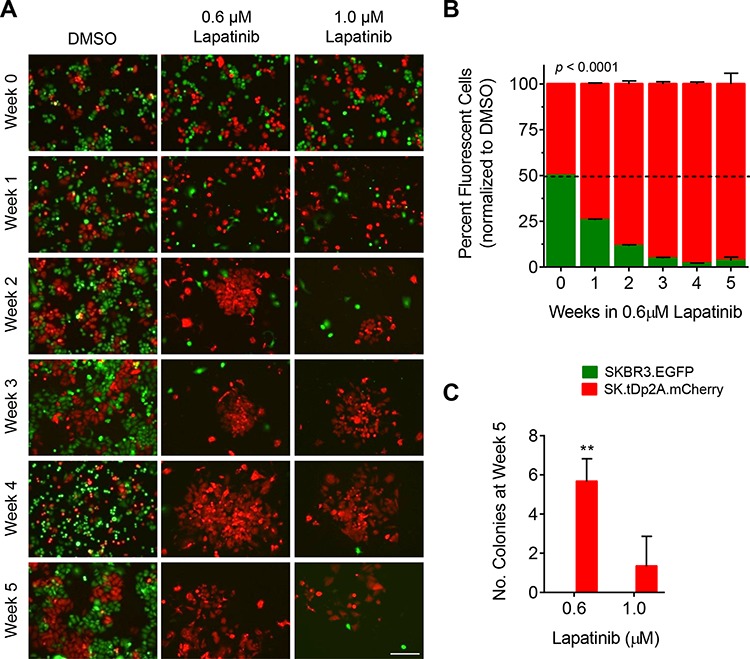
Colony formation by SK.tDp cells exposed to lapatinib SKBR3 cells stably expressing EGFP (SKBR3.EGFP) and SK.tDp cells stably expressing mCherry (SK.tDp2A.mCherry) were co-cultured at a ratio of 1:1 and continuously exposed to 0.1% DMSO, 0.6 μM lapatinib or 1.0 μM lapatinib for 5 weeks. DMSO-treated cells were split twice-weekly (1:4 dilution). Lapatinib-treated cells were grown without passaging. Each experiment was run in triplicate. **A.** Co-cultured cells were imaged weekly for fluorescence (10x magnification, scale bar = 200 μm). Shown are representative fields for each condition and time point. **B.** The percentage of EGFP-positive and mCherry-positive cells in each co-culture was quantitatively measured weekly via flow cytometry. Data was normalized to the mean percentage of EGFP-positive and mCherry-positive cells, respectively, in DMSO-treated co-cultures; mean ± standard deviation. **C.** For each co-culture the number of fluorescent colonies was counted after 5 weeks in lapatinib; mean ± standard deviation, **p ≤ 0.01.

To attribute the survival advantage in lapatinib directly to t-Darpp overexpression, the previous experiment was repeated with 1:1 co-cultures of SKBR3.EGFP and SK.tDp2A.mCherry cells. Similar results were observed, but with a faster progression. SK.tDp colony formation in lapatinib was observed as early as two weeks in drug (Fig. 5A) and a predominance of mCherry-positive cells was observed by flow cytometry after only one week in lapatinib (Fig. 5B). This trend continued for the following four weeks. By week 5, mCherry-positive cells were the predominant cell type (*p* < 0.0001) and the predominant colonies (*p* = 0.001) in the lapatinib co-cultures (Fig. 5B and 5C). These results were verified in non-fluorescent SK.tDp cells to rule out any effect of mCherry itself (Supplementary Fig. 5).

## DISCUSSION

HER2-targeted drugs are important components of breast cancer therapy, both as frontline agents in the case of trastuzumab and as secondary options when trastuzumab resistance emerges. Finding either unique or common mechanisms of resistance to HER2-targeted therapies could help predict therapeutic response and also might reveal additional targets for therapeutic intervention in combination with currently available drugs. t-Darpp was first discovered as a cancer-specific alternative form of Darpp-32, a well-known effector of PKA signaling in neuronal cells. t-Darpp expression tends to increase while Darpp-32 expression decreases during malignant transformation and tumor progression, both in humans and mice [18, 26], and there have been multiple reports demonstrating that t-Darpp overexpression confers resistance to trastuzumab and other drugs [7–10]. We have previously reported that Darpp-32 and t-Darpp have antagonistic roles in determining a cell's sensitivity to trastuzumab [8]. The shift in the relative amounts of Darpp-32 and t-Darpp in lapatinib-resistant cells (Fig. 1) suggests that this ratio, with a need for excess t-Darpp, might play a role in the development of lapatinib resistance as well.

Consistent with the lack of immediate cross-resistance to lapatinib in trastuzumab-resistant patients, t-Darpp does not appear to confer outright resistance to lapatinib as determined by a relatively short-term cell proliferation assay (Fig. 2B). t-Darpp does appear to confer a survival and/or growth advantage in lapatinib, however, as evidenced by the more rapid emergence of lapatinib-resistant colonies for SK/Her^R^ and SK.tDp cells (Fig. 4 and 5). This emphasizes the importance of the assay in distinguishing between an endpoint resistance phenotype and what could be an early stage of progression to resistance. Indeed, if we want to understand what is happening in the clinic, it might be necessary to study the process of acquiring resistance as well as the end product. In the case of lapatinib, the drug was initially approved because trastuzumab-resistant patients respond positively to treatment, but it is becoming clear that secondary resistance to lapatinib develops quickly, thus rendering the drug somewhat ineffective overall [3, 5]. Our results provide a possible mechanism by which trastuzumab-resistant tumors might develop lapatinib resistance – by dysregulating the apoptotic process – even after an initial response to the therapy.

It is interesting to speculate about the mechanism by which t-Darpp could prevent BIM upregulation or accumulation in response to lapatinib (Fig. 3). We know from the current work that t-Darpp must be exerting its effect independently of Akt, since Akt phosphorylation is completely inhibited in response to lapatinib in cells that overexpress t-Darpp (Supplementary Fig. 1). It is possible that t-Darpp exerts its effect via protein kinase A (PKA). Several groups, including our own, have shown that t-Darpp upregulates PKA activity [8, 27, 28]. PKA is capable of phosphorylating FoxO3a, a transcriptional regulator of BIM, independent of the PI3K pathway [29, 30]. Such phosphorylation would promote FoxO3a degradation and prevent it from stimulating BIM transcription. PKA is also capable of phosphorylating BIM directly, although there are conflicting reports on whether this phosphorylation has a stabilizing or de-stabilizing effect [31–33]. Further experimentation will resolve the question of whether t-Darpp acts via PKA, which could in turn prevent BIM transcription or promote BIM degradation. In either case, the end result would be a failure to accumulate BIM to levels required for apoptosis [25, 34, 35]. Other mechanisms are possible and there could be multiple mechanisms leading to the same end result in lapatinib-resistant patients. Nevertheless, our findings suggest that t-Darpp is one factor that, when present, can play a role in resistance to lapatinib and to other drugs that rely on BIM upregulation to induce apoptosis.

## MATERIALS AND METHODS

### Cell culture and reagents

The human HER2+, estrogen receptor-negative breast cancer cell line, SKBR3 and a SKBR3-derived trastuzumab-resistant cell line, SK/Her^R^, were kindly provided in 2009 by Rita Nahta (Emory University, Atlanta, GA) [36, 37]. The derivation of SK.empty (SK.e) and SK.tDp cells was described previously [8]. Briefly, SKBR3 cells obtained in 1999 from the American Type Culture Collection (Rockville, MD) were transfected with an empty pcDNA3.0/Neo vector (SK.e) or with the same vector containing full-length t-Darpp cDNA (SK.tDp). Cells were transfected used Lipofectamine 2000 (Invitrogen Life Technologies) according to the manufacturer's instructions and stably transfected cells were selected and maintained in 1 mg/mL G418. Selection conditions for lapatinib-resistant SK/Lap^R^ cells are described in the Results and Discussion section. The origins of SKBR3, SK/Her^R^, SK/Lap^R^ 2.0 I.P and transfected SK.tDp cell lines were verified in 2014 by the ATCC/Promega Cell Authentication Service. SKBR3 and SKBR3-derived cells were maintained in McCoy's Medium 5A with 10% FBS, 1% penicillin/streptomycin and 1% L-glutamine in 5% CO_2_. SK/Her^R^ and SK/Lap^R^ cells were maintained in drug at their original selection concentrations (4 mg/mL trastuzumab and 2 μM lapatinib, respectively). Trastuzumab (Genentech San Francisco, CA) and lapatinib (GlaxoSmithKline, Research Triangle Park, NC) were obtained from the City of Hope National Medical Center Pharmacy (Duarte, CA). Trastuzumab was reconstituted in Bacteriostatic Water for Injection. Lapatinib ditosylate monohydrate was purified from the medicinal tablet via chromatography and diluted in dimethyl sulfoxide (DMSO). A minimum of five days prior to each experiment all drugs were removed from the culture medium to allow for drug clearance from cells.

### Drug sensitivity assays

CellTiter-Glo luminescence assay: Cells were plated at a density of 4 × 10^4^ cells per well in 96-well plates. The following day, media containing either 0.1% DMSO or 2 μM lapatinib was added. After 5 days of drug treatment, cell number was determined by performing the Promega CellTiter-Glo Luminescence Assay (Madison, WI) according to the manufacturer's instructions. Luminescence was measured using a Veritas Microplate Luminometer (Turner BioSystems, Sunnyvale, CA). Data are presented as relative cell number and normalized to the luminescence of DMSO-treated cells. Each experiment was run in quadruplicate and repeated a minimum of three times. Sulforhodamine B (SRB) assay: SRB assays were performed as previously described [8]. Cells were treated with media containing either 0.1% DMSO or lapatinib at concentrations ranging from 0.01–10 μM for 5 or 7 days before analysis. Each experiment was run in quadruplicate and repeated a minimum of three times.

### Western analysis

Cell lysates were collected on ice in SDS Lysis Buffer from EMD Millipore (Billerica, MA) supplemented with 1X protease inhibitor cocktail from Roche Applied Science (Indianapolis, IN). Protein concentrations were determined by RC DC protein assay purchased from Bio-Rad Laboratories (Hercules, CA). Proteins were separated on either a 12% SDS-PAGE gel or a precast 4–12% gradient NuPAGE^®^ Bis-Tris gel from Life Technologies (Grand Island, NY) and then transferred to a nitrocellulose membrane. 5% non-fat dry milk was used for blocking buffer and for primary antibody incubation. Primary antibodies: an antibody that recognizes both Darpp-32 and t-Darpp (#H62) from Santa Cruz Biotechnology (Santa Cruz, CA); antibodies to α-Tubulin (#T5168) and β-Actin (#A4700) from Sigma-Aldrich Corporation (St. Louis, MO); an antibody to Survivin (#NB-500–201) from Novus Biologicals (Littleton, CO); and antibodies to BIM (#2933), pHER2 (Y1248, #2243), total HER2 (#4290), pEGFR (Y2234, #2234), total EGFR (#2242), pAkt (S473, #4058), total Akt (#4058), pERK (T202/Y204, #4377) and total ERK (#9102) from Cell Signaling Technology (Danvers, Massachusetts). Secondary antibodies were horseradish peroxidase-conjugated anti-mouse IgG and anti-rabbit IgG antibodies from Cell Signaling Technology. Secondary antibody was detected using an ECL Plus kit from Thermo Fisher Scientific. Protein expression was quantified using ImageJ software and expressed as relative density, normalized to loading control values. Each experiment was repeated a minimum of two times.

### RNA preparation and RT-PCR

Total RNA was isolated and purified using the Qiagen RNeasy kit (Valencia, CA). RNA was reverse transcribed to cDNA using random primers and SuperScript III Reverse Transcriptase from Life Technologies. Darpp-32 and t-Darpp mRNA levels were analyzed by quantitative RT-PCR (1 cycle of 3 min at 95°C, 40 cycles of 10 sec at 95°C, 30 sec at 60°C, and a melting curve 55–95°C) using the PerfeCTa^®^ SYBR^®^ Green SuperMix from Quanta BioSciences (Gaithersburg, MD). Each experiment was run in triplicate and repeated a minimum of three times. Primers 5′-CCGCAAGAAGATCCAGTTCTCGGT-3′ and 5′-CTCCTCTGGTGAGGAGTGCTCTGA-3′ were used to measure Darpp-32 mRNA; primers 5′-TGCGCTGGCTCAGTCTCCTTC-3′ and 5′-GGGA GGCTTCCTCCTCTGGTGAG-3′ were used to measure t-Darpp mRNA; and primers 5′-GAGAAGGCTGGGG CTCATTTGC-3′ and 5′-GTTGGTGGTGCAGGAGG CATTG-3′ were used to measure GAPDH mRNA.

### siRNA transfection

Cells were plated in 60-mm dishes at a density of 2.5 × 10^5^ cells per dish using media without penicillin/streptomycin. The following day, cells were transiently transfected with 60 pmol of siRNA using Lipofectamine RNAiMAX from Invitrogen by Life Technologies according to the manufacturer's instructions. Control siRNA against GFP (5′-GCUGACCCUGAAGUUCAUCUG-3′) and a pool of three target-specific siRNA against Darpp-32 and t-Darpp from Santa Cruz Biotechnology (#sc-35173) were used. 48 hours post-transfection, media containing 0.1% DMSO or 2 μM lapatinib was added for a period of 24 hours when cell lysates were collected for Western analysis.

### Caspase-Glo 3/7 apoptosis assay

Cells were plated at a density of 4 × 10^4^ (SKBR3) or 1 × 10^5^ (SK/Lap^R^ 2.0 I.P) cells per well in a 96-well plate. A higher initial cell number was used for SK/Lap^R^ cells because they are highly sensitive to transfection with siRNA (data not shown). The following day cells were transfected with 2 pmol of siRNA (see siRNA transfection methods above). After a 48 hour exposure to 0.1% DMSO or 2 μM lapatinib, the number of cells undergoing apoptosis was quantified using the Promega Caspase-Glo 3/7 Assay kit according to the manufacturer's instructions. Data are presented as relative apoptosis and normalized to the luminescence of siCtrl, DMSO-treated cells for each cell line. Each experiment was run in triplicate and repeated a minimum of two times.

### Stable transfections

To establish cell lines stably expressing fluorescent markers, we subcloned EGFP (enhanced green fluorescent protein) cDNA and mCherry cDNA into the pcDNA3.0/Neo vector. pcDNA.EGFP/Neo and pcDNA.mCherry/Neo were transfected into SKBR3 and SK/Her^R^ cells, respectively, using Lipofectamine LTX/PLUS (Invitrogen by Life Technologies) according to the manufacturer's instructions. Stably transfected populations (SKBR3.EGFP and SK/Her^R^.mCherry) were selected and maintained in 1 mg/mL G418. To make the pcDNA.tDp/mCherry/Neo vector, t-Darpp cDNA with a 2A peptide sequence [38] attached to the C-terminus was subcloned into the pcDNA.mCherry/Neo vector upstream of and in-frame with the mCherry sequence. The plasmid was then transfected into SKBR3 cells and the stably transfected population (SK.tDp2A.mCherry) was selected and maintained in 1 mg/mL G418. Each transfected cell population was fluorescently sorted via flow cytometry (using the Aria II SORP cell sorter from Becton Dickinson, Franklin Lakes, New Jersey) to isolate the cell populations with the highest fluorescent protein expression. Sorted cells were characterized as described in the Results and Discussion section and then used in all subsequent co-culture experiments.

### Fluorescent cell co-culture experiments

Fluorescent cell co-cultures were plated at a ratio of 1:1 (EGFP:mCherry) at a density of 3 × 10^5^ cells per well in 6-well plates (for fluorescence imaging) or 1.5 × 10^6^ cells per dish in 10-cm dishes (for flow cytometry). Cells were incubated with 0.1% DMSO, 0.6 μM lapatinib or 1.0 μM lapatinib. DMSO-treated cells were split twice weekly, whereas media for cells grown in lapatinib was changed weekly. Experiments were run in triplicate and repeated a minimum of two times. Live cell fluorescent imaging and colony counts: For co-cultures maintained in 6-well plates, fluorescence was imaged weekly using a Zeiss AxioVert 200 Inverted microscope with a Zeiss Mr3 camera and 10X/0.3NA EC Plan-Neofluar objective using Zeiss AxioVision 4.8 software. At the conclusion of the experiment (five weeks in drug), the number of EGFP- and mCherry-fluorescent colonies per well was counted. For non-fluorescence supplemental experiments, cells were imaged in bright field and stained with methylene blue for the purpose of counting colonies. Flow cytometry: For co-cultures maintained in 10-cm dishes, the numbers of EGFP- and mCherry-positive cells were determined weekly. Triplicate plates of cells were trypsinized, washed with cold PBS and suspended in ice cold DNase Buffer (PBS, 1% FBS, 100 units/mL DNase, 1 mM MgCl_2_) containing 0.5 μg/mL DAPI. A Fortessa SORP (Becton Dickinson) analytical cytometer with DiVa 6.1.3 software was used to quantitatively analyze fluorescence. 50,000 events were measured per sample, and DAPI-positive cells were excluded from the analysis.

### Statistical analysis

Statistically significant differences were calculated using the GraphPad Prism 6.0 statistical program. Differences between groups were determined by the two-tailed Student's *t*-test or the two-way ANOVA. *p* values < 0.05 were considered significant.

## SUPPLEMENTARY FIGURES


