# Supplementary material for: t-Darpp overexpression in HER2-positive breast cancer confers a survival advantage in lapatinib
Source: Oncotarget. 2015 Sep 28;6(32):33134–45. doi: 10.18632/oncotarget.5311 (PMC4741754; doi:10.18632/oncotarget.5311)
Supplement: Supplementary file 1 [file oncotarget-06-33134-s001.pdf]

SUPPLEMENTARY FIGURES

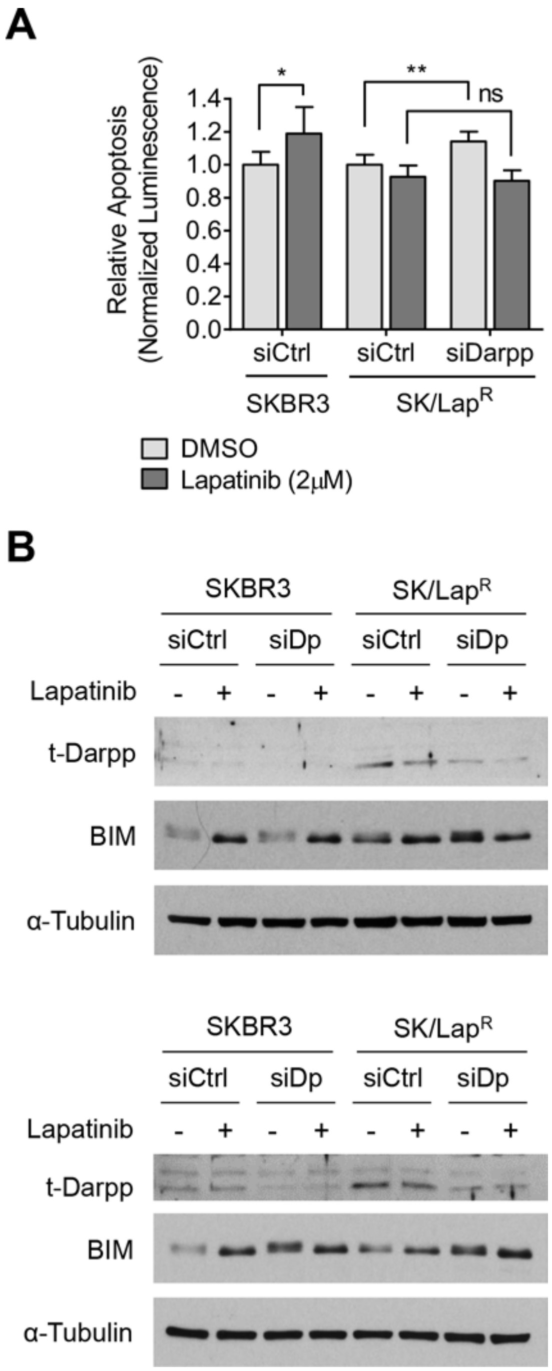

Supplementary Figure S1: t-Darpp down-regulation in lapatinib-resistant cells. SKBR3 and SK/Lap^{R} 2.0 I.P cells were transiently transfected with siRNA targeted to GFP (siCtrl) or Darpp-32/t-Darpp (siDp). **A.** The relative levels of apoptosis were determined by Caspase-Glo 3/7 assay after 48 hour exposure to 0.1% DMSO or 2 μM lapatinib. Data was normalized to the mean luminescence of siCtrl, DMSO-treated cells. Bars represent mean ± standard deviation from two separate experiments, six total replicates. *$p \leq 0.05$, **$p \leq 0.01$. **B.** Western analysis of t-Darpp and BIM protein levels after 24 hour exposure to 0.1% DMSO (−) or 2 μM lapatinib (+). Data are from two separate experiments run in parallel with the apoptosis assays. α-Tubulin was used as a loading control.

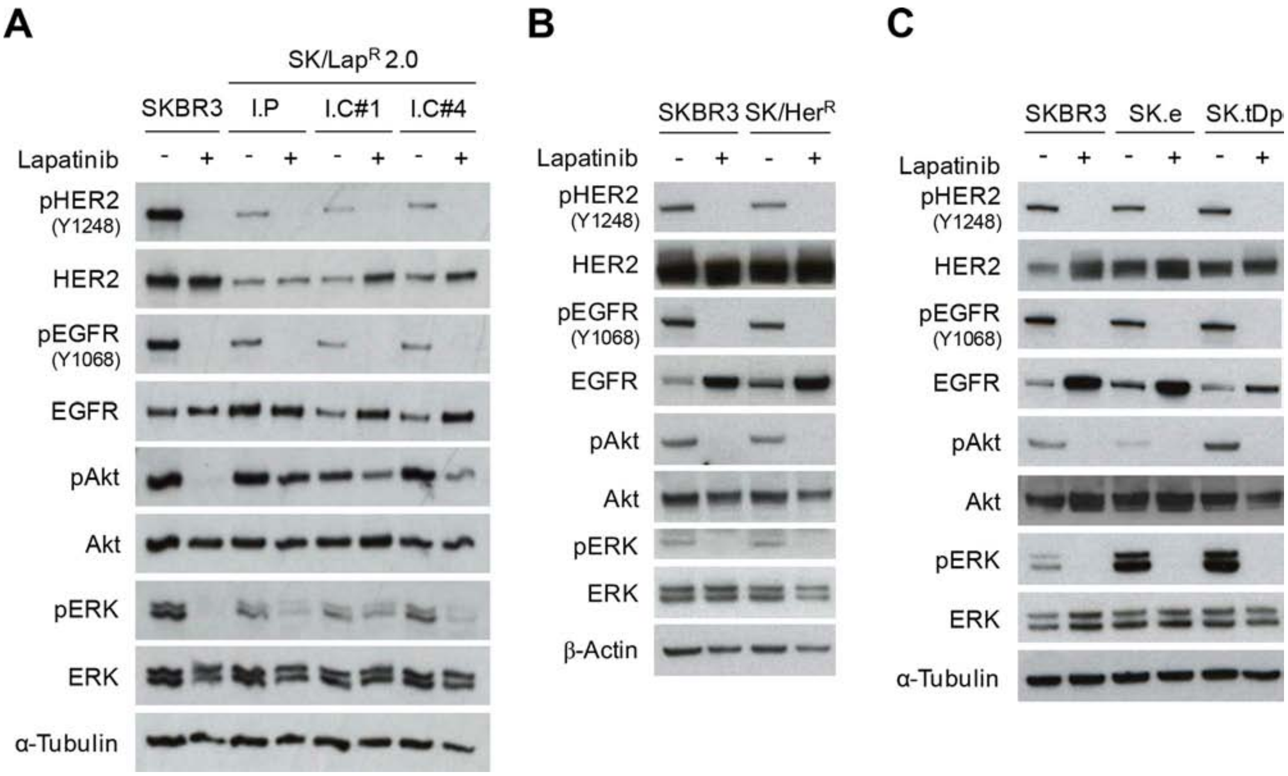

Supplementary Figure S2: Cell signaling in response to lapatinib. Western analysis of phosphorylated and total protein levels in cells exposed to 0.1% DMSO (−) or 2 μM lapatinib (+) for 24 hours. α-Tubulin and β-Actin were used as loading controls. **A.** Protein expression in lapatinib-resistant SK/Lap^R cell lines. **B.** Protein expression in trastuzumab-resistant SK/Her^R cells that overexpress endogenous t-Darpp. **C.** Protein expression in stably transfected SK.tDp cells that overexpress exogenous t-Darpp.

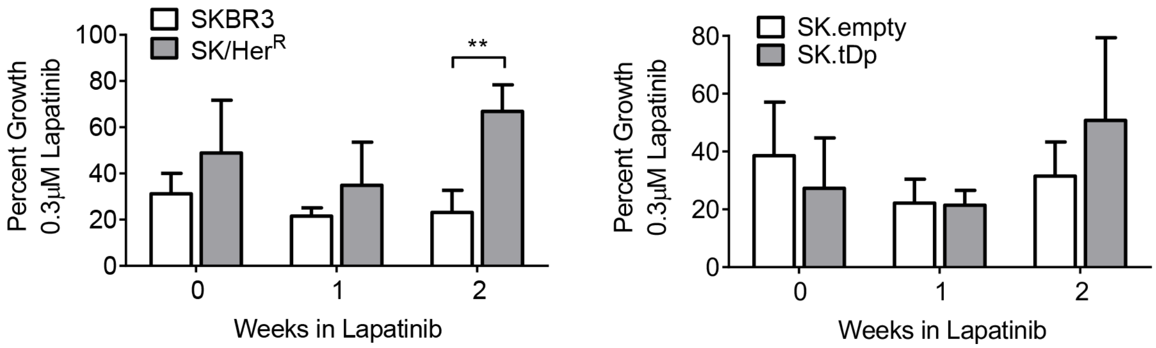

Supplementary Figure S3: Changes in lapatinib sensitivity in cells overexpressing t-Darpp. SK/Her^R and SK.tDp cells were exposed continuously to 0.3 µM lapatinib for 2 weeks. Each week cell sensitivity to lapatinib was retested by SRB assay. Data was normalized to the mean absorbance of DMSO-treated cells; mean ± standard deviation, **$p \leq 0.01$.

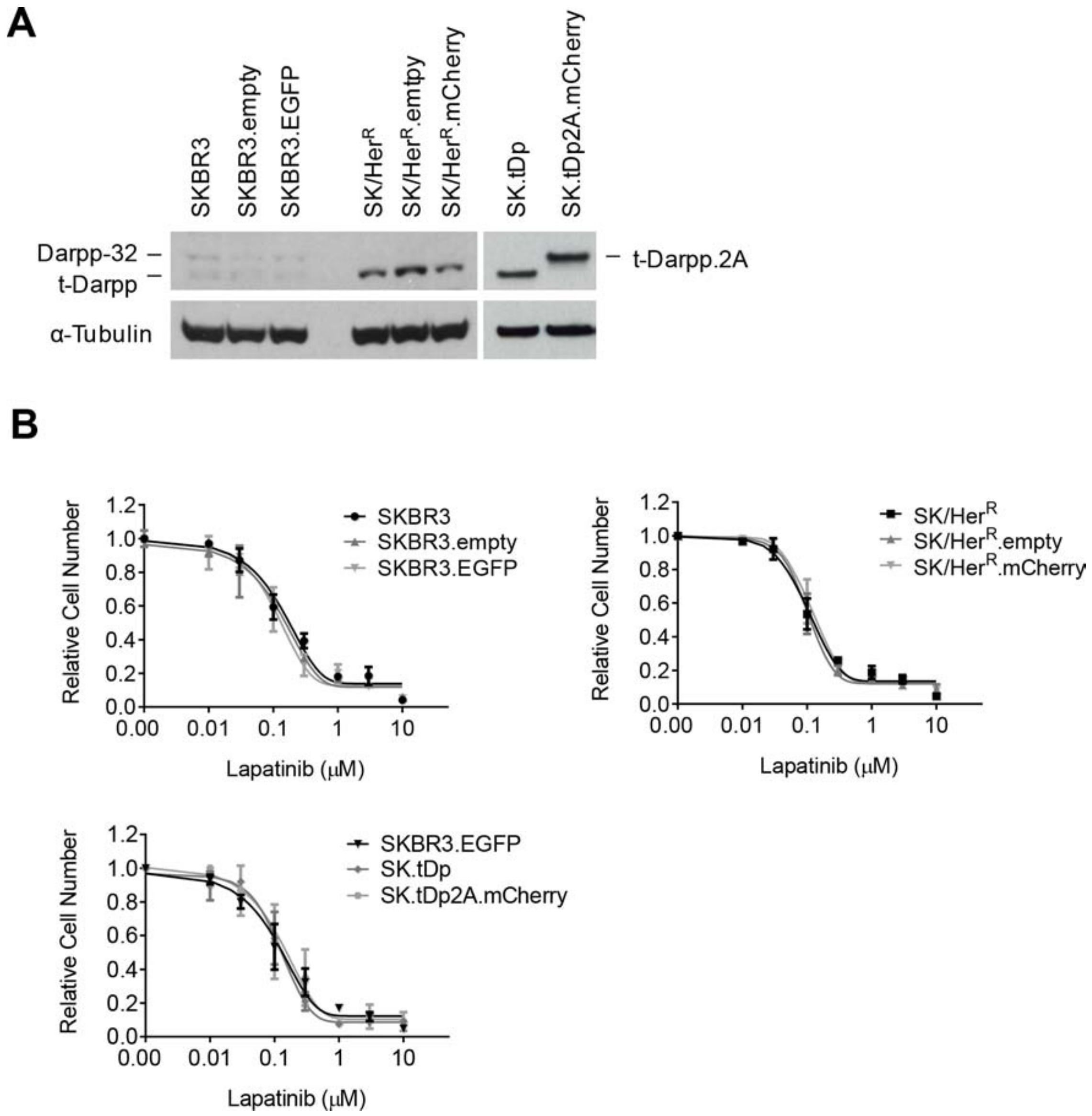

Supplementary Figure S4: Characterization of stably transfected fluorescent cell lines. A. Darpp-32 and t-Darpp protein levels were measured by Western analysis. α-Tubulin was used as a loading control. **B.** Proliferation in lapatinib was quantified by SRB assay after 5-day exposure to 0.1% DMSO or increasing concentrations of lapatinib. Data was normalized to the mean absorbance of DMSO-treated cells; mean ± standard deviation.

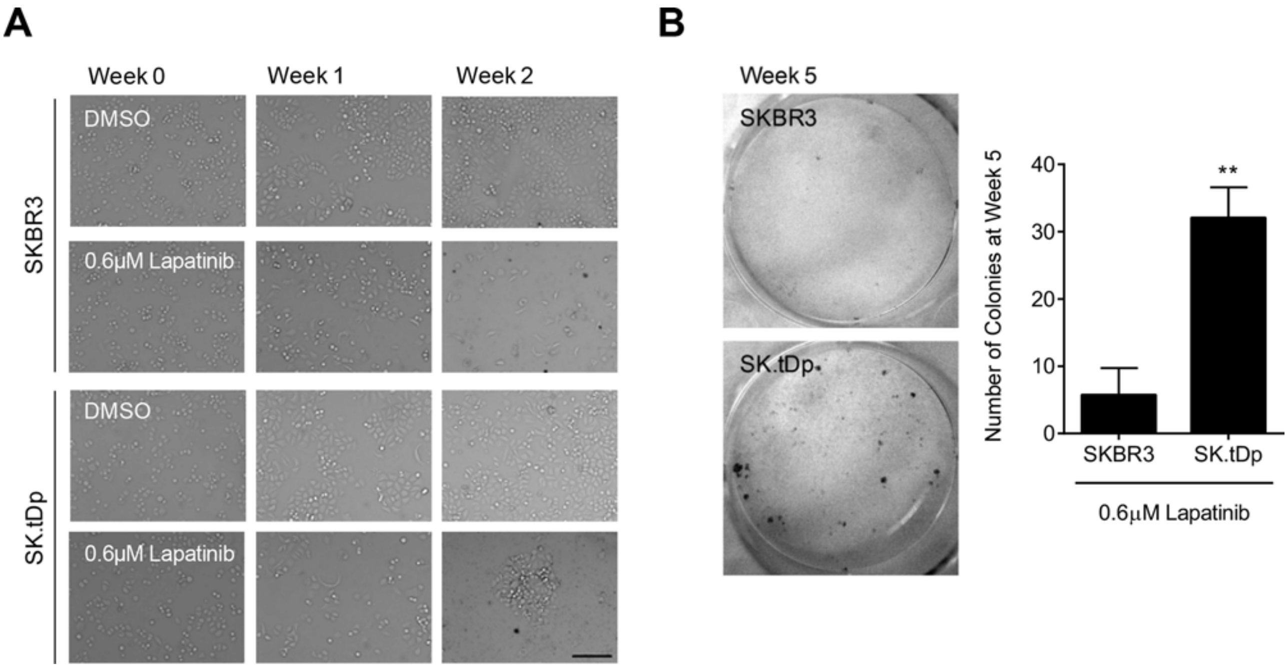

Supplementary Figure S5: Colony formation by non-fluorescent SK.tDp cells exposed to lapatinib. SKBR3 or SK.tDp cells were continuously exposed to 0.1% DMSO or 0.6 μM lapatinib for 5 weeks. DMSO-treated cells were split twice-weekly (1:4 dilution). Lapatinib-treated cells were grown without passaging. Each experiment was run in triplicate. **A.** Cells were imaged weekly for 2 weeks (10x magnification, scale bar = 200 μm). Shown are representative fields for each condition and time point. **B.** After 5 weeks of lapatinib exposure cells were fixed and stained with methylene blue. A representative well is shown for each cell line exposed to lapatinib. The number of colonies in triplicate wells was counted; mean ± standard deviation, $**p \leq 0.01$.
